# The Bidirectional Relationship Between Sleep and Inflammation Links Traumatic Brain Injury and Alzheimer’s Disease

**DOI:** 10.3389/fnins.2020.00894

**Published:** 2020-08-25

**Authors:** Tabitha R. F. Green, J. Bryce Ortiz, Sue Wonnacott, Robert J. Williams, Rachel K. Rowe

**Affiliations:** ^1^BARROW Neurological Institute at Phoenix Children’s Hospital, Phoenix, AZ, United States; ^2^Department of Child Health, University of Arizona College of Medicine – Phoenix, Phoenix, AZ, United States; ^3^Department of Biology and Biochemistry, University of Bath, Bath, United Kingdom; ^4^Phoenix Veteran Affairs Health Care System, Phoenix, AZ, United States

**Keywords:** sleep, inflammation, traumatic brain injury, concussion, Alzheimer’s disease, cytokines, microglia, neurodegeneration

## Abstract

Traumatic brain injury (TBI) and Alzheimer’s disease (AD) are diseases during which the fine-tuned autoregulation of the brain is lost. Despite the stark contrast in their causal mechanisms, both TBI and AD are conditions which elicit a neuroinflammatory response that is coupled with physical, cognitive, and affective symptoms. One commonly reported symptom in both TBI and AD patients is disturbed sleep. Sleep is regulated by circadian and homeostatic processes such that pathological inflammation may disrupt the chemical signaling required to maintain a healthy sleep profile. In this way, immune system activation can influence sleep physiology. Conversely, sleep disturbances can exacerbate symptoms or increase the risk of inflammatory/neurodegenerative diseases. Both TBI and AD are worsened by a chronic pro-inflammatory microenvironment which exacerbates symptoms and worsens clinical outcome. Herein, a positive feedback loop of chronic inflammation and sleep disturbances is initiated. In this review, the bidirectional relationship between sleep disturbances and inflammation is discussed, where chronic inflammation associated with TBI and AD can lead to sleep disturbances and exacerbated neuropathology. The role of microglia and cytokines in sleep disturbances associated with these diseases is highlighted. The proposed sleep and inflammation-mediated link between TBI and AD presents an opportunity for a multifaceted approach to clinical intervention.

## Introduction

In diseased states affecting the central nervous system (CNS), the fine-tuned autoregulation of the brain is lost, which contributes to the affective and cognitive symptoms seen in psychiatric, neurological, and mental health disorders (Charrier et al., 2017). Alzheimer’s disease (AD) is a neurodegenerative disorder that impacts approximately 1 in 10 people over the age of 65 and 1 in 3 people over the age of 85 (Newcombe et al., 2018). AD has a long prodromal phase and progresses slowly, across a span of typically around 10 years. In contrast, traumatic brain injury (TBI) is a rapid onset condition which can occur at any age due to impact to the head. It has been suggested that TBI heightens the risk of subsequent development of AD (Mendez, 2017). Despite the stark contrast in their causal mechanisms, both AD and TBI are conditions which elicit a neuroinflammatory response that is coupled with physical, cognitive, and affective symptoms. A commonly reported symptom in both AD and TBI is sleep disturbance (Vitiello and Borson, 2001; Castriotta et al., 2007; Viola-Saltzman and Watson, 2012; Sandsmark et al., 2017). Sleep is regulated by circadian and homeostatic processes, such that pathological inflammation may disrupt the chemical signaling required to maintain a healthy sleep profile. In this way, immune system activation can influence sleep physiology. Conversely, sleep disturbances can exacerbate symptoms or increase the risk of inflammatory/neurodegenerative diseases. The coupling between sleep and inflammation may indicate clinical disease status or outcome of disease processes (Wiseman-Hakes et al., 2013; Rao et al., 2014). The self-perpetuating cycle of sleep disturbance and neuroinflammation seen in TBI and AD encourage a comparison of these seemingly disparate conditions to gain greater insight into the shared mechanisms of progressive symptomology. This review will discuss the possibility of a sleep- and inflammation-fueled progression from TBI to AD.

**FIGURE 1:**
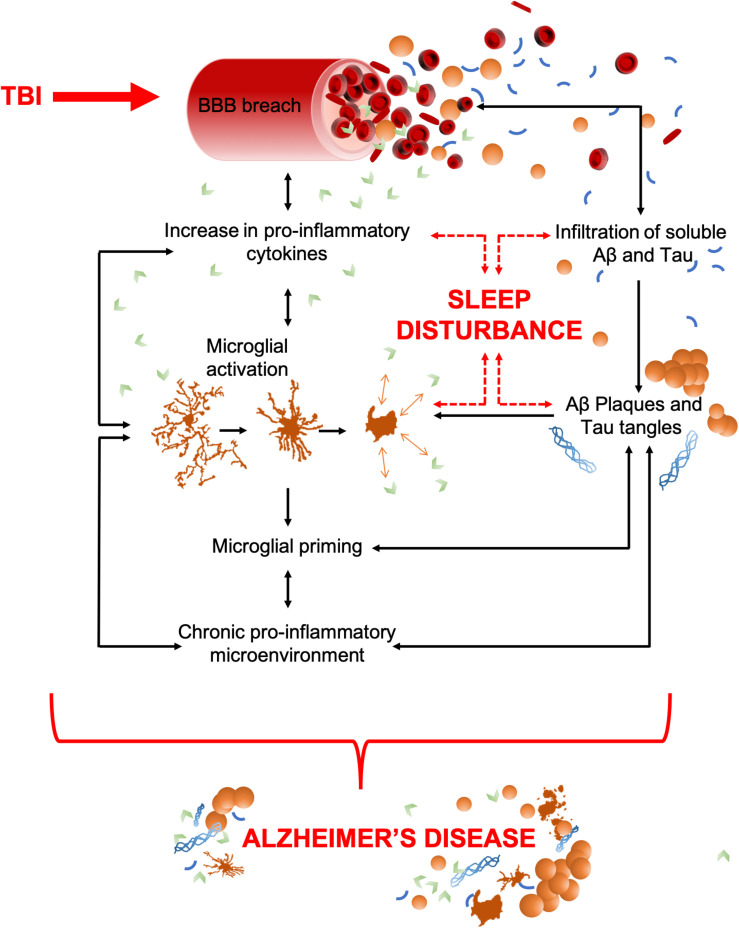
TBI to AD, an inter-disease trajectory. TBI disrupts the blood brain barrier (BBB) upon insult which results in an infiltration of peripheral pro-inflammatory cytokines and any soluble pools of amyloid-β (Aβ) and Tau. Together, these can precipitate AD. As some pro-inflammatory cytokines have dual (opposing) roles as sleep regulatory substances, their increase can also lead to sleep disturbances, a characteristic that commonly precedes the cognitive decline in AD. Pro-inflammatory cytokines upregulate the activation of microglia, which act as a positive feedback mechanism, resulting in increased pro-inflammatory cytokine production and an increased breach of the BBB. Unregulated cytokine release also sustains microglial activation and priming which results in a chronic pro-inflammatory microenvironment. This includes astrocytosis, hypoxia, reactive oxygen species (ROS), elevated cytokine levels, and microglial activation. The movement of amyloid-β (Aβ) and Tau through the breach in the BBB could potentially seed protein oligomerization and aggregation, thereby acting as possible drivers of central plaque and tangle pathology. Such aggregates in the brain further contribute to microglial activation, the pro-inflammatory microenvironment, and neuronal apoptosis. Together, these contribute to cognitive dysfunction and brain atrophy, the key pathological features of AD. Both brain atrophy and neuronal death help to sustain the pro-inflammatory microenvironment creating a self-perpetuating feedback loop.

TBI is characterized by a primary insult, initiated by mechanical forces applied to the head or brain. Immediately, sequential pathophysiological processes are initiated, which often result in a chronic inflammatory microenvironment. Microglia and resident mononuclear phagocytes throughout the CNS are activated as part of this secondary injury process in an attempt to preserve homeostasis and repair injured tissue. TBI-induced inflammation is communicated by pro- and anti-inflammatory cytokines, which are below detectable levels in healthy tissue but rapidly increase upon impact (Wang and Shuaib, 2002; Lucas et al., 2006). Cytokines are powerful chemical communicators, essential for maintaining homeostasis throughout the body. However, unregulated release of pro-inflammatory cytokines following an injury can cause pathological functions that lead to detrimental inflammation and progressive tissue damage (Kumar and Loane, 2012; Morganti-Kossmann et al., 2018). Cytokines released by microglia act as sleep regulatory substances (SRSs) and help to maintain healthy sleep (Krueger and Majde, 1995). Therefore, TBI-induced elevation of cytokines can lead to sleep disturbances. Hereon, cytokine-mediated inflammatory cascades and sleep enter a self-perpetuating positive-feedback loop, containing many of the components seen in the molecular circuitry of sleep disturbances in AD (as shown in Figure 1).

AD, much like TBI, is associated with a range of cognitive and non-cognitive symptoms that include memory loss, disturbed sleep, speech difficulties, depression, and loss of executive function (Burns and Iliffe, 2009). Preceding cognitive decline, the aggregation of amyloid-β plaques and tau neurofibrillary tangles occurs in the brain, both of which are required for a retrospective AD diagnosis after death (Murphy and LeVine, 2010). While a minority of cases are genetically linked, most do not have an identified cause which makes AD hard to predict, characterize, diagnose, and treat (Newcombe et al., 2018). Preexisting diseases and lifestyle factors can also contribute to an increased risk of AD (Faden and Loane, 2015). These include TBI, a common injury which increases the frequency and onset of AD and other related dementias (McKee et al., 2009; Gavett et al., 2010; Gavett et al., 2011; Baugh et al., 2012). Similar to TBI, AD has a neuroinflammatory component that is orchestrated by pro-inflammatory cytokines and activated microglia. Although inflammation has a beneficial role in clearing cellular debris and apoptotic cells associated with AD, chronic inflammation can be damaging due to pro-inflammatory intermediates that compromise future clearance mechanisms, synaptic pruning, and neuronal survival (Solito and Sastre, 2012; Sarlus and Heneka, 2017). Moreover, the cytokine storm associated with chronic inflammation can disrupt sleep-wake cycles, a symptom frequently observed in AD patients. Sleep disturbances cause further cytokine release and microglial activation, hence a positive feedback loop of chronic inflammation and sleep disturbance is initiated (as shown in Figure 1).

In this review, we discuss the bidirectional relationship between sleep disturbance and inflammation, such that chronic inflammation associated with TBI and AD can lead to sleep disturbances that exacerbate neuropathology. We also highlight the role of microglia and cytokines in sleep disturbances associated with these diseases.

## The Role of Sleep on Homeostasis

While sleep is an evolutionarily conserved phenomenon that is essential for survival (Banks and Dinges, 2007), its function is not fully understood. The roles of sleep in the elimination of waste, restoration of depleted energy sources (Oswald, 1980; Dworak et al., 2010), and energy conservation (Walker and Berger, 1980; Xie et al., 2013) have long been characterized. Sleep plays a physiological role in the cardiovascular, metabolic, thermoregulatory, respiratory, and sexual systems, as well as having a major role in regulating brain health and function. More recent hypotheses suggest a cellular need for sleep in regulating brain plasticity (Puentes-Mestril and Aton, 2017), learning, and memory (Tononi and Cirelli, 2003; Huber et al., 2004; Tononi and Cirelli, 2014; Miyamoto et al., 2017; Raven et al., 2018). Indeed, in the absence of sleep, there is significant detriment to cognitive function (Krueger et al., 1999).

The sleep-wake cycle is essential in regulating many of the body’s fine-tuned homeostatic processes, even on a molecular level (Cirelli and Tononi, 2008). A study, using two-photon imaging of live mice after infusion of fluorescent tracers, showed that during sleep there was a 60% increase in the interstitial space, allowing an increase in exchange between cerebrospinal fluid (CSF) and interstitial fluid. This has important implications for the clearance of interstitial disease-associated proteins (Xie et al., 2013) and highlights a role for sleep in maintaining brain homeostasis.

## TBI – Inflammation and Sleep Pathology

Mechanical forces applied to the head or brain initiate TBI, wherein sequential pathophysiological processes permanently change neurological function (Masel and DeWitt, 2010; Corrigan and Hammond, 2013). TBI survivors suffer irreversible cognitive, sensory, sleep, mental health, and emotional morbidities as a consequence of injury-induced pathological processes (McAllister, 1992; Kempf et al., 2010). TBI can cause neuronal cell death, ischemia, hemorrhage, and the disruption of the blood-brain barrier (BBB) which elicit neuroinflammatory cascades (Pleines et al., 2001). Such secondary injury can exacerbate the damage caused by the initial impact if left unresolved. Although the primary brain injury from TBI is irreversible, the subsequent injury processes occur in a delayed fashion and may be responsive to treatment.

Ongoing cellular events post-TBI often cause further damage and lead to physiological consequences (Werner and Engelhard, 2007; Prins et al., 2013). Among these, sleep disturbances after TBI are commonly reported in the acute timeframe post-injury by up to 70% of TBI survivors (Cohen et al., 1992; Orff et al., 2009). Sleep disturbances may persist chronically (Castriotta et al., 2007; Verma et al., 2007; Kempf et al., 2010), and are experienced across the spectrum of TBI patients, including children and adolescents (Tham et al., 2012). These sleep disturbances in TBI survivors ultimately impact their quality of life. Identification and treatment of sleep-wake disturbances following TBI can improve outcomes in vigilance, working memory, and capacity of language processing (Wiseman-Hakes et al., 2013). Excessive daytime sleepiness is the most common sleep-wake disturbance reported among TBI patients (Castriotta et al., 2007; Kempf et al., 2010; Baumann, 2012) and is characterized primarily by an increase in sleep propensity. Other commonly reported disorders include post-traumatic hypersomnia, narcolepsy, delayed sleep phase, insomnia, and fatigue (Ouellet and Morin, 2006; Verma et al., 2007; Kempf et al., 2010; Baumann, 2012; Billiard and Podesta, 2013). These sleep disturbances negatively impact rehabilitation of TBI patients and can exacerbate symptoms such as pain and cognitive deficits (Mathias and Alvaro, 2012; Bhalerao et al., 2013).

Extensive pre-clinical research has focused on the detrimental effects of inflammation on the injured brain. In rodent models of TBI, microglia respond immediately to brain injury-induced tissue damage and release inflammatory mediators such as inflammatory cytokines and chemokines that have been shown to alter sleep (Morganti-Kossmann et al., 2001; Davalos et al., 2005; Nimmerjahn et al., 2005; Frugier et al., 2010; Semple et al., 2010; Ziebell and Morganti-Kossmann, 2010; Bachstetter et al., 2013). Two-photon microscopy images of fluorescently labeled microglia following a laser-induced injury demonstrated rapid proliferation and migration of microglia to the site of injury, where their processes fused (Davalos et al., 2005). It was hypothesized that this fusion event was to create a barrier between healthy and injured tissue (Davalos et al., 2005). These findings suggest that microglia may be the first line of defense following TBI (Kumar and Loane, 2012). However, microglia can become over-activated and induce detrimental neurotoxic effects through the overproduction of cytotoxins, including cytokines (Block and Hong, 2005). Uncontrolled cytokine production by activated microglia can significantly influence activation of astrocytes, which can increase neuronal cell death and worsen outcomes after TBI (Myer et al., 2006). Together, these results support the view that the inflammatory response to TBI possesses both beneficial and detrimental effects that likely differ in the acute and delayed phase after injury.

In the mouse, diffuse brain injury has been shown to increase the parameters of sleep for 6 h post-injury, regardless of sex, injury severity, or time of day the injury occurs (Rowe et al., 2014b; Saber et al., 2019). This period of post-traumatic sleep correlates with elevated central and peripheral cytokine levels, particularly SRSs. Subsequent studies have shown sleep impairment at light-dark transitions, suggesting possible TBI-induced disruption of circadian rhythms (Lim et al., 2013; Rowe et al., 2014a). Whether post-traumatic sleep is beneficial or detrimental to neurological outcome from TBI remains unresolved. To advance the understanding of post-traumatic sleep, a gentle handling approach was used to keep both uninjured and brain-injured mice awake over the 6 h of post-traumatic sleep. In this study, a single period of sleep deprivation demonstrated no adverse, long-standing neurological effects (Rowe et al., 2014a), although, the course of recovery from injury may have been altered. Three days of transient sleep disruption following experimental TBI in the mouse worsened inflammation and altered stress-immune pathways (Tapp et al., 2020). Total sleep deprivation for 24 h following diffuse TBI in rats reduced morphological damage and improved functional outcome in the acute recovery period (Martinez-Vargas et al., 2012). The effect of sleep deprivation prior to TBI has also been investigated and 48 h of sleep deprivation or chronic sleep restriction (10 days, 6-h sleep/day) prior to mild or moderate TBI in rats did not exacerbate brain injury-induced neuronal damage (Caron and Stephenson, 2015). Sleep after TBI has also been inhibited pharmacologically. The therapeutic treatment of mice with an anti-inflammatory lipid mediator, and treatment with a novel TNF-α inhibitor, attenuated TBI-induced sleep and subsequently improved functional outcome (Harrison et al., 2015; Rowe et al., 2018b). When a second TBI was introduced during post-traumatic sleep (3 h apart), more severe functional and histopathological outcomes were observed, when compared with mice that received a second TBI after sleep from the initial impact subsided (Rowe et al., 2018a). Taken together, these studies suggest that more evidence is needed to determine if acute sleep after TBI is beneficial or detrimental, however, the underlying pathological response to brain injury that results in increased sleep may be amenable to therapies. Furthermore, post-traumatic sleep may be a physiological response to brain injury that could serve as a personalized biomarker to pathological conditions. In moderate to severe clinical cases of TBI, sleep-wake cycles can be altered due to a loss of consciousness. Clinical data indicate when the brain has not recovered consciousness from a moderate to severe TBI, it is not able to consolidate sleep or generate a 24-h sleep-wake cycle (Duclos et al., 2017). These clinical data parallel experimental data and suggest in the acute phase of TBI, injury-induced pathophysiology contributes to sleep disturbances.

Although inflammation has emerged as a major player in sleep disturbances post-TBI, there are other influences that should be considered. TBI can cause widespread mechanical damage to regions of the brain, many of which exert a role in sleep and inflammation. One region of particular interest to understanding sleep disturbances post-TBI is the hypothalamus (Sundaram et al., 2013). Damage or loss of hypothalamic neurons can disrupt orexin (a.k.a. hypocretin) secretion which has a well-established role in arousal and wakefulness. Extensive loss of orexin neurons and consequent excessive daytime sleepiness have been observed both pre-clinically and clinically after TBI (Baumann et al., 2009; Thomasy et al., 2017; Thomasy and Opp, 2019). Experimental TBI in the mouse decreased wake and increased non-rapid eye movement (NREM) sleep during the dark period and reduced orexin cells as a function of injury severity up to 2 weeks post-injury (Thomasy et al., 2017). Orexin (hypocretin) knockout mice subjected to TBI did not exhibit altered sleep, whereas control wild type mice had increased NREM sleep and decreased wake up to 1 month post-TBI (Thomasy and Opp, 2019). A prospective clinical study found that TBI survivors who reported excessive daytime sleepiness had reduced CSF orexin levels up to 6 months post-injury (Baumann et al., 2007). Together, these data support that the orexinergic system may be involved in long-term sleep-wake alterations that persist after the acute inflammatory response to TBI has subsided. Therefore, orexins should be considered when discussing the sleep-mediated links between TBI and AD (Liguori, 2017). However, the complexity of this relationship must be noted as not only has increased orexinergic signaling been associated with the progression of AD, orexins have been shown to increase amyloid-β in the interstitial fluid (Kang et al., 2009) and there is a documented correlation between tau proteins and orexin CSF levels and sleep-wake dysregulation in AD patients (Liguori et al., 2020b). Furthermore, damage to the hypothalamus and pituitary can affect the endocrine system in general (Sundaram et al., 2013), with potential for further impact on sleep architecture that is currently poorly understood.

## Dysregulated Immune Function and Sleep

The immune system is the body’s defense system which monitors, detects, and attempts to eliminate threats to health and homeostasis. Immune cells communicate via chemical mediators such as cytokines and chemokines allowing them to respond to pathological changes within tissues with a high degree of specificity. While the peripheral immune system plays a role in the disease progression of TBI and AD, the inflammatory reactions observed in these conditions are largely driven by microglia, the immune cells exclusive to the CNS. There are bidirectional links between sleep and the immune system, such that sleep loss impairs immune function and physiological sleep is modified in response to an immune challenge (Ingiosi and Opp, 2016). Sleep/circadian disturbances, particularly states of sleep disruption and deprivation, can lead to serious consequences; for example, dysregulation of the inflammatory response (Castanon-Cervantes et al., 2010; Besedovsky et al., 2019), leading to a state of systemic inflammation with increased pro-inflammatory cytokines in the brain (Marshall and Born, 2002; Opp and Toth, 2003; Maurovich-Horvat et al., 2008; Aldabal and Bahammam, 2011).

### Cytokines and Sleep

Cytokines have an essential role in regulating the immune system at multiple stages throughout the body and have long been characterized as a contributing factor to sleep and sleep disturbances. Under normal conditions, cytokines have been shown to play a role in NREM sleep (Shoham et al., 1987; Morrow and Opp, 2005; Krueger, 2008), as well as altering neuronal firing patterns in both the hypothalamus and brainstem, with consequent effects on sleep regulation (Opp, 2005). Interleukin (IL)-6, tumor necrosis factor (TNF)-α, and IL-1β, are well characterized for their essential role in both sleep and inflammation and mediate the crosstalk between the peripheral immune system and the brain (Krueger and Majde, 1995; Krueger, 2008; Besedovsky et al., 2019). Mechanistically, this property of IL-6, TNF-α, and IL-1β, could be linked to their neuromodulatory effect in contributing to the fine-tuned control of synaptic transmission and plasticity (Jewett and Krueger, 2012; Vezzani and Viviani, 2015), which contributes to the function of memory formation during intermittent rapid eye movement (REM) sleep and NREM sleep (Walker, 2017). As such, increased production of these pro-inflammatory cytokines mediates increases in sleep under inflammatory conditions such as TBI or AD.

There is also a relationship between sleep disturbances and cytokine production [Please see (Irwin, 2019) for an excellent review on sleep and cytokines]. The sleep disturbances that occur in neurological disease and injury (Musiek and Holtzman, 2016; Leng et al., 2019; Logan and McClung, 2019) are likely due to common pathological pathways that activate the immune response; specifically, inflammation and cytokine production. Prolonged wakefulness increased cytokines in the CSF (Borbely and Tobler, 1989; Krueger et al., 2001), which can lead to subsequent sleep disturbances. In one study, REM sleep deprivation for 72 h in rats led to significant increases in plasma IL-6, IL-1β, and TNF-α expression, but the expression of IL-6 and IL-1β returned to levels of controls 1-week after the period of sleep deprivation ended, while TNF-α remained elevated (Yehuda et al., 2009). Moreover, sleep deprivation increased IL-1β and TNF-α mRNA expression in cortical and subcortical brain regions in rats (Zielinski et al., 2014). Similarly, in humans with experimental chronic circadian sleep disruption, there was a significant increase in plasma TNF-α expression (Wright et al., 2015). A meta-analysis also showed that in humans, sleep disturbances increased plasma IL-6, although no significant changes were found in TNF-α expression, likely owing to the low statistical power in the analysis (Irwin et al., 2016). These studies support a bidirectional relationship between these pro-inflammatory cytokines and sleep. Increases in these cytokines lead to increased sleep, and conversely, sleep deprivation and disruption can increase these cytokines. However, a recent study showed that TNF-α knockout mice display similar patterns of sleep and no deficiency in sleep regulation compared with wild type mice (Szentirmai and Kapás, 2019). In fruit flies, knockdown of the TNF-α homologue in astrocytes, but not neurons, reduced sleep duration and disrupted sleep-rebound following sleep deprivation (Vanderheyden et al., 2018). As such, although IL-6, IL-1β, and TNF-α promote sleep, these cytokines may have a larger role in regulating sleep following inflammatory insults such as after TBI or during the chronic inflammatory events of AD.

Moreover, cytokines have functional implications in both TBI and AD. Following a TBI, there is a profound increase in the production and release of cytokines (Figure 1). Acutely, the increased release of cytokines is believed to have therapeutic implications that can help improve primary and secondary injury (Gyoneva and Ransohoff, 2015; Plesnila, 2016). However, chronically elevated cytokine levels can further perpetuate the initial damage and lead to robust secondary damage (Morganti-Kossman et al., 1997; Zeiler et al., 2017). Similarly, in AD, a dysregulation of cytokines may potentiate the pathogenesis of the disease leading to irreversible damage (Su et al., 2016; Figure 1). Indeed, plasma cytokines are higher in the AD population when compared with healthy controls (Swardfager et al., 2010). In the context of this review, we assert that sleep disturbances, brought about by either TBI or AD, lead to changes in cytokine production and may further exacerbate disease-related damage to the brain.

## Alzheimer’s Disease – Inflammation and Sleep Pathology

Sleep disturbances have long been a symptom associated with AD but more recently have been recognized as an early feature of the disease, thought to precede cognitive decline and contribute to disease progression (Musiek et al., 2015; Musiek and Holtzman, 2016). Literature reports that 25–70% of all suspected AD patients report disturbances in sleep which are linked to poorer disease prognosis (Moran et al., 2005; Craig et al., 2006; Beaulieu-Bonneau and Hudon, 2009; Lim et al., 2014; Wennberg et al., 2017). A recent study found that 20% of people between 25 and 45 were sleeping 90 min less than the recommended amount for their age group (Léger et al., 2011). This raises the question as to whether irregular sleep in early life contributes to developing AD later in life.

Sleep is important for clearing pathological and inflammatory-associated proteins from the brain. The recently discovered glymphatic system clears unwanted or pathological proteins from the interstitial space in the brain by exchange between the CSF the interstitial fluid (ISF) (Xie et al., 2013). Aquaporin-4 water channels, located on the end feet on astrocytes, mediate the process by allowing ISF flow from the interstitial space into the paravenous region, which in turn drains through the lymphatic system (Verheggen et al., 2018). This is the process by which solutes are removed from the brain. Further, it has been shown that the level of amyloid-β in the brain fluctuates rapidly with the daily sleep-wake cycle. During sleep, there is a 60% increase in the flow of ISF to CSF. Therefore, it is postulated that the disturbed sleep of AD patients decreases the exchange of ISF and CSF which may lead to reduced clearance of soluble amyloid-β, contributing to the formation of plaques (Xie et al., 2013; Rasmussen et al., 2018). In turn, increased amyloid burden can lead to an upregulated level of inflammation, perpetuating the cycle. Clinically, this effect has been observed using accelerated neuroimaging; blood oxygen level, functional magnetic resonance imaging, electroencephalogram, and CSF flow. Using these techniques, waves of CSF flow were observed during slow wave sleep, which appeared to be coupled with the hemodynamic rhythm during sleep (Fultz et al., 2019). Further, a recent clinical study that examined the sleep architecture and CSF biomarkers (tau and amyloid-β_42_) in patients with AD (which progressed from having subjective cognitive impairment to mild cognitive impairment to AD), found that both REM and NREM sleep were disturbed. They also found that AD patients displayed a decrease of CSF amyloid-β_42_ (Liguori et al., 2020a). In the clinic, amyloid-β deposition has been shown to accumulate prior to cognitive and memory decline (Liguori et al., 2020a) and this effect was significantly higher in those who also had poor sleep efficiency (Molano et al., 2017). Other experimental studies on human volunteers have also suggested that dysregulated sleep patterns increase amyloid-β deposition in the human brain (Shokri-Kojori et al., 2018). Together, these data demonstrate how the self-perpetuating cycle of sleep disturbance and inflammation are associated with a decline in cognitive function.

**FIGURE 2:**
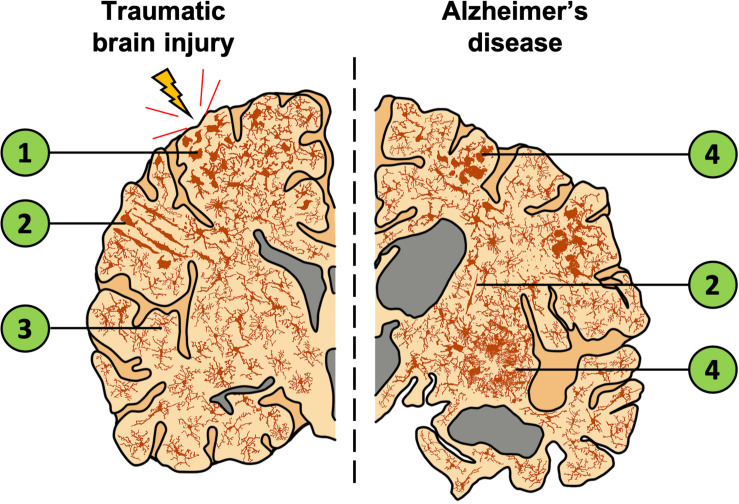
Depiction of a coronal brain slice showing the global expanse of microglial phenotypes in TBI and AD. The left illustrates localized effects of TBI which include increased microglial activation near the injury site and decreased activation in distal regions. In comparison, the right represents the AD brain with widespread changes in microglia morphology, gross structural changes to the cortex, and an enlargement of ventricles (gray). Both TBI and AD lead to increased inflammation and activated microglia. Despite this similarity, distinct microglial morphologies are observed in these conditions. (1) Microglia are activated, migrate to the injury site, and display an amoeboid or phagocytic morphology. (2) Rod-cell morphology is often observed in the cortex after TBI and have also been documented in AD tissue. However, the function of this cell phenotype is currently unknown, and the morphology is not restricted to TBI/AD or the region in which they are shown. (3) Distal to the injury site, microglia are ramified and occur at a lower density. (4) Microglia that surround amyloid-β plaques show activated, amoeboid, and dystrophic morphologies. Cells of each phenotype pictured are not restricted to the brain regions shown.

Pre-clinically, transgenic mice overexpressing disease-associated APPswe/PS1δE9 mutations which drive amyloid-β pathology, exhibit disturbed sleep patterns prior to developing amyloid-β plaques (Roh et al., 2012). This disturbance further emphasizes the potential bidirectional relationship between amyloid-β deposition and sleep regulation. However, whether the detrimental cycle is between sleep and amyloid-β directly, or rather associated inflammatory factors (or a combination of both) is unknown. The extent to which amyloid-β acts as a driver of AD progression remains controversial, but it does appear very likely that inflammation increases amyloid-β deposition (Irwin and Vitiello, 2019). Clinically, the AD brain presents with activated microglial phenotypes, most notably aggregated in the penumbra of amyloid-β plaques (Navarro et al., 2018) (see Figure 2; further discussed in section “TBI as a Contributor to AD”), where their likely/presumed function is to reduce neuronal exposure to soluble amyloid β-mediated toxicity (Wang et al., 2016). In the post-mortem AD brain, other morphological changes in microglia are seen, such as rod and dystrophic phenotypes, as well as an overall increase in microglial density (Figure 2; Bachstetter et al., 2015). This suggests that the role of microglia in AD is probably more complex than simply clearing amyloid-β and associated proteins (as represented in Figure 2). Despite the elusiveness of their function, these altered microglial phenotypes are associated with an increased release of pro-inflammatory cytokines known to cause sleep disturbances and neuronal toxicity (Lull and Block, 2010), thus, through this mechanism microglia can contribute to the progression of AD. In summary, both pre-clinical and clinical research suggests a complex interaction between sleep and the microglia-mediated inflammatory response in the AD brain.

Both sleep and inflammation are tightly coupled to the cholinergic system, which promotes wakefulness and REM sleep (Platt and Riedel, 2011). Irreversible damage to the basal forebrain cholinergic system correlates with the memory and cognitive symptoms seen in AD (Ferreira-Vieira et al., 2016). Moreover, the immune response is in part regulated by the cholinergic anti-inflammatory pathway by control of macrophages and microglia through α7 nicotinic receptors. Such modulation attenuates the release of pro-inflammatory cytokines, namely TNF-α, as shown *in vitro* and *in vivo* (Shytle et al., 2004; Lehner et al., 2019) and might be compromised by the loss of acetylcholine from basal forebrain neurons in AD. Although the acetylcholinesterase inhibitors donepezil, galantamine, and rivastigmine (drugs currently licensed to treat the symptoms of AD) would be anticipated to boost acetylcholine levels, they have a poorly defined effect on sleep architecture (Cooke et al., 2006). One study found an 81.8% improvement in sleep quality with galantamine treatment, 75% with rivastigmine, and 50% from donepezil (Naharci et al., 2015). Despite these reported improvements, the treatment of sleep disturbances in AD patients remains a clinical challenge. Glutamatergic signaling, which plays an integrated role in the regulation of sleep stages and arousal (Shi and Yu, 2013), also deteriorates in AD patients. Memantine, a moderate affinity N-methyl-D-aspartate receptor antagonist, has been shown to increase total time spent sleeping in AD patients treated with 20 mg/day for 4 weeks (Ishikawa et al., 2016). However, due to the nature of receptor desensitization and transient efficacy of this drug, it is not a good long-term pharmacological candidate to break the altered sleep architecture and inflammation cycle discussed here. In the search for a more effective intervention, multiple neuroinflammatory molecules have been targeted as potential treatments for AD but have had limited success in measurable outcomes. Currently, there is renewed interest in microglia as a therapeutic target to stop the pathological inflammatory cascades seen in AD which could then potentially treat sleep disturbances, as discussed in section “TBI to AD – A Microglial Mediated Progression?”.

### TBI as a Contributor to AD

Meta-analyses have found that a history of TBI is associated with development of AD (Mortimer et al., 1991; Fleminger et al., 2003). Despite different mechanisms of onset, these conditions are mechanistically interlinked by a complex web of pathologies, as demonstrated by Figure 1. Furthermore, increased amyloid-β levels are seen after TBI (Sivanandam and Thakur, 1995; Rasmusson et al., 1995; Nemetz et al., 1999; Jellinger et al., 2001) and post-mortem examination of TBI brains found that amyloid-β plaques, a hallmark of AD, had developed acutely in 30% of TBI brains (Roberts et al., 1991; Roberts et al., 1994; Johnson et al., 2010). However, as amyloid-β plaques are also seen in the healthy brain (Rodrigue et al., 2009), the meaning of these findings are unclear. While there is an increased risk of AD after TBI, most individuals with AD do not have a history with TBI, thus, other factors may interact with TBI-induced damage and lead to exacerbated cognitive decline and dementia (Plassman and Grafman, 2015). As discussed above, sleep disturbances post-TBI correlate with microglial activation and a concomitant rise in pro-inflammatory cytokine levels (Rowe et al., 2014b). As sleep disturbances have been identified as an early symptom of AD, it is plausible that TBI-induced sleep disturbances are a risk factor for AD. Until this is understood, the opportunity for therapeutic intervention in this inter-disease period is stunted.

### Breach of the Blood Brain Barrier – Unregulated Infiltration of Plasma Proteins

TBI, despite the varying causal mechanisms, initiates breach of the BBB. Whether it be a transient opening due to the shearing of blood vessels, or a chronic inflammation-mediated opening, cross-talk between the CNS and the blood can occur. This is due to disruption of the tight junctions that prevent uncontrolled infiltration of substances from the blood, as summarized by Figure 1. A scanning electron microscopy (SEM) study of the human brain after death from TBI showed altered vascular features (Rodríguez-Baeza et al., 2003). Microvascular casts examined by SEM showed a sunken vascular surface, longitudinal creases, and flattened luminal morphology (Rodríguez-Baeza et al., 2003). A similar breach in neurovascular membranes has been observed in many pre-clinical studies (Tanno et al., 1992; Baldwin et al., 1996; Sangiorgi et al., 2013). Not only does the initial brain injury cause BBB breach from the sheering caused by the mechanical force exerted on the brain, but secondary processes such as inflammation and hypoxia contribute to prolonged breach of the BBB creating a self-perpetuating, unregulated cycle of cross-talk between the CNS and the blood. Alongside TBI, BBB breach is a well-known consequence of many other inflammation-inducing conditions such as viral or bacterial infection. Amyloid-β has also been shown to cause cerebral-amyloid angiopathy (the presence of amyloid-β aggregates in the vasculature of the brain) which adds to the perpetual, unregulated infiltration of blood components into the brain as this also causes further loss of blood vessel integrity leading to brain hemorrhage (Smith and Greenberg, 2009). As both mechanical damage and infiltration of pro-inflammatory molecules that travel in the blood increase the inflammatory microenvironment after TBI, this may increase the likelihood of developing AD, and its consequent sleep disturbances, that are known to be tightly associated with inflammation.

Matrix metalloproteases (MMPs) are key enzymes that cause breakdown of BBB integrity, and therefore the infiltration of products into the brain (Nissinen and Kähäri, 2014). MMP9 knockout mice have shown the deleterious role of MMPs in maintaining a chronic ischemic environment after a transient, focal ischemic injury (Asahi et al., 2001), as the absence of MMP9 reduced proteolytic breakdown of the BBB. Further, MMPs increase the bioavailability of both TNF-α (Haro et al., 2000; Vandenbroucke et al., 2013) and IL-1β (Schönbeck et al., 1998; Nissinen and Kähäri, 2014), two major inflammatory cytokines that can cause breach of the BBB independent of injury (Siegel et al., 2009). Not only does this lead to a chronic pro-inflammatory environment of the brain, but TNF-α and IL-1β act as SRSs, the increase of which can cause sleep disturbances. The disruption of sleep patterns feeds into the upregulation of inflammation and is a key feature of AD which can precede cognitive decline. When considered together, this body of literature suggests that MMPs are mediators of the perpetual pathological feedback loop initiated by TBI and postulated to increase the risk of developing AD.

### Amyloid-β and Tau as Mediators of Inflammation and Sleep Disturbance

It is well established that TBI can cause disruption to the BBB, henceforth, crosstalk between the CNS and peripheral organ systems is initiated. Not only can this allow infiltration of peripheral cytokines (which further exacerbate neuroinflammation), but soluble forms of amyloid-β and tau can move across the BBB. The following section will discuss the role of these proteins in the inflammatory mediated sleep disturbances seen during the progression of both TBI and AD.

There is little known about the brain-wide concentration of oligomeric and unaggregated forms of amyloid-β, a protein that is found in high abundance peripherally with respect to the brain. As TBI creates an opportunity for amyloid-β proteins to cross the BBB, the clinical and biochemical impact of this is under much dispute. One study examined patients with a severe TBI and found that amyloid-β peptides (amyloid-β 1–42 and 1–40) were increased in the CSF, peaking 1 week post-injury (Raby et al., 1998). Increased levels of tau and amyloid-β were also observed in blood exosomes (Gill et al., 2018). Such increases in these proteins may be reflected in increased levels in CSF, however, evidence has proven inconsistent. For example, two studies reported that amyloid-β in the CSF was significantly lower than in controls during the week following TBI (Franz et al., 2003; Kay et al., 2003). Despite the disparity of these findings, it could be interpreted that CSF amyloid-β decreased post-injury due to possible deposition in the brain. However, as sleep disturbance post-TBI peaks in the acute time phase, and sleep disturbance increases soluble amyloid-β in the CSF (Lucey et al., 2018), the findings of these studies are conflicting. Moreover, researchers have shown that the anti-inflammatory antibiotic doxycycline can attenuate the acute behavioral deficits associated with intracerebral delivery of oligomeric amyloid-β (Forloni and Balducci, 2018), which acts as proof of principle; unaggregated forms of amyloid-β and inflammation are tightly coupled.

Not only does infiltration of amyloid-β and tau directly affect the formation of plaques and tangles and increase oligomeric toxic species, but infiltration of associated proteins can also have an effect. For example, S100A9 (a pro-inflammatory protein) has been shown to contribute to amyloid plaques that accumulate rapidly after TBI. These plaques were found to be positive for oligomeric forms of amyloid-β and not fibrillar forms, suggesting that these could be a precursor to the plaques seen in later stage AD (Wang et al., 2014). Further, it has been well accepted that the levels of iron, copper and zinc are altered in the brain of AD patients and that amyloid-β plaques are sites of metal aggregation. Experimental models of TBI have also shown that there is increased infiltration of metal ions (iron, copper and zinc) post-TBI. Under normal physiological conditions, such ions are found in the blood and are prevented from freely moving across the BBB. Amyloid-β has been shown to associate with zinc, copper and iron ions (Lovell et al., 1998), which are all present in the blood, therefore, the increased level of blood in the brain after TBI is likely to exacerbate this association. This increase in ion concentration is therefore likely to augment plaque formation, increasing plaque-associated inflammation. Another well characterized contributor to AD is oxidative stress caused by the rapid production of free radicals. As sleep fosters antioxidant activity, sleep disruption may exacerbate ROS activity and associated inflammation post-TBI, which can lead to neuronal cell death and further aggregation of amyloid-β (Molina-Holgado et al., 2007).

TBI can also augment the formation of amyloid-β plaques and tau neurofibrillary tangles (NFTs) through inflammation-dependent gene expression and transcription factor activation. Experimental TBI in the 3xTg mouse model of AD, showed that TBI activated transcription factors (CCAAT/Enhancer Binding Protein Beta), and increased the expression of delta-secretase (Wu et al., 2019). Delta-secretase is an enzyme that mediates pathology by cleaving amyloid-β and tau which allows the formation of amyloid-β plaques and hyperphosphorylated tau, and consequently induces the neuroinflammatory cascade (Zhang et al., 2020). Support for the hypothesis that TBI augments the formation of amyloid-β plaques and NFTs was strengthened by the fact that the authors reversed the effect using viral expression of tau in 3xTg mice, that was resistant to cleavage by delta secretase (Wu et al., 2019). These data demonstrate how amyloid- β and tau contribute to the inflammatory microenvironment (and consequent sleep disturbances) associated with both TBI and AD.

NFTs are another hallmark of AD. In both mice and humans, NFTs have been found post-TBI (Kondo et al., 2015) and play a critical role in post-injury neuronal damage (Smith et al., 1999; Duda et al., 2000; Uryu et al., 2007; Yao et al., 2008; Johnson et al., 2012). NFTs impair axonal transport, notably of mitochondria (which are necessary for supporting nerve terminal function), contributing to the neuronal death associated with TBI (Kondo et al., 2015). Tau acts intracellularly as a microtubule stabilizer; however, its accumulation can occur both intracellularly and extracellularly (Swanson et al., 2017). Intracellularly tau abnormalities may cause neuronal death through disruption of intracellular cargo transport, whereas extracellular oligomers of tau are released from degenerating neurons. This was shown by in-vitro studies, revealing addition of un-aggregated tau protein increased the amount of cell death in tau-treated cells compared to controls (Gómez-Ramos et al., 2006). This suggests that extracellular tau is able to transmit the pathology to neighboring neurons, which causes spread of neuronal death from tauopathy (Swanson et al., 2017). Further, tau induces synaptic loss through microglial phagocytosis of synaptic compartments containing tau, leading to synaptic loss and cognitive decline (Jadhav et al., 2015). Alongside mediating the spatiotemporal spread of pathology, tau can prolong microglial activation, exacerbating a chronic pro-inflammatory environment in the brain, as shown in Figure 2.

A recent study using positron emission tomography has identified an increased level of tau deposition in patients that had suffered a single, moderate-severe TBI compared to controls (Gorgoraptis et al., 2019). Pre-clinical rodent models have also found an increase in oligomeric tau in the brain acutely (4 h) after parasagittal fluid percussion injury, which remained elevated for 2 weeks post-injury compared to uninjured shams (Hawkins et al., 2013). These studies support an increase in tau protein in the brain after TBI and provides another link for an inter-disease progression to AD triggered by TBI-induced BBB disruption.

Not only has tau been shown to have inflammatory consequences, but a recent human study, using regression analysis on positron emission tomography measures of amyloid-β and tau, and EEG sleep recording, showed that the extent of slow wave sleep disturbance predicted a greater presence of tau in the medial temporal lobe (Winer et al., 2019). Numerous studies using animal models also associate aggregates of tau with disturbance to sleep or circadian regulation (Musalek et al., 1989; Witton et al., 2016; Holth et al., 2017; Arnes et al., 2019). Taken together the data presented in this section suggest a plausible inter-condition link between TBI, AD through the inflammatory and sleep mediated pathological cycle.

## TBI to AD – A Microglial Mediated Progression?

Microglia, the immune cells exclusive to the CNS (Loane and Byrnes, 2010), play a role in perpetuating homeostasis in the CNS (Inoue, 2002), where they act as a biological surveillance system continuously monitoring the microenvironment. This allows them to be the first responders upon detection of pathological stimuli (Glenn et al., 1992). Microglial activation displays a morphological (and genetic) continuum, with changes in characteristics enabling the cells to rapidly respond to stimuli. Under normal conditions, microglia have a small cell soma and highly branched processes. After detection of pathological stimuli, or sleep disturbance, the cell soma enlarges, and the processes retract. This activated phenotype permits phagocytic activity (Liu and Quan, 2018). Upon activation, microglia release cytokines to trigger pro-inflammatory pathways. Despite some immediate benefit seen from microglial activation, such as clearing of cellular debris, their reactivity has been associated with potentially damaging intermediates. These include reactive oxygen species and pro-inflammatory cytokines (Norden and Godbout, 2013; Iizumi et al., 2016), which can cause damage to nearby neurons, prolong the inflammatory response, hinder CNS repair, and exacerbate neurological symptoms, such as inflammation-induced sleep (Dheen et al., 2007; Ziebell and Morganti-Kossmann, 2010; Bilbo and Stevens, 2017). Further, chronic microglial activation increases astrocyte activation, which can worsen the outcome after an inflammatory challenge, increasing neuronal cell death (Myer et al., 2006).

In response to pathological stimuli, microglia upregulate their release of certain cytokines, including TNF-α, IL-1β, and IL-6 which act as SRSs (see section “Cytokines and Sleep”). A further consideration is that higher levels of microglial activation are observed during waking hours; microglia return to their non-activated status during sleep (Fonken et al., 2015). As microglia upregulate their release of IL-1β and TNF-α upon activation, it is likely that these cytokines fluctuate in response to endogenous circadian regulation, though this is beyond the scope of current experimental literature. If microglia return to non-activated states during sleep, one would predict sleep is neuroprotective following pathological stimuli, however, these data are inconclusive and require further research (see section “Alzheimer’s Disease – Inflammation and Sleep Pathology”).

The inflammatory cascade present in both AD and TBI presents as an attractive target for therapeutic intervention. Historically, cyclooxygenase, a key enzyme in prostaglandin production, was identified as a target for reducing the inflammatory response seen in AD, however, its inhibition with nonsteroidal anti-inflammatory drugs (Aisen, 2002) had limited efficacy in stalling the progression of AD. Due to observed clustering of microglia around amyloid-β plaques, microglia have emerged as a therapeutic target. Despite the growing volume of multidisciplinary research on the activation of microglia and pro-inflammatory cytokines as SRSs, the nuances of their involvement in sleep are not yet fully understood. As dysregulation of sleep has been associated with neurological deficits, a complex, multifaceted feedback loop is created. This poses a difficult target for therapeutic intervention, until the role of microglia in sleep is fully unveiled.

Multiple areas of neuroscientific research have presented cogent evidence suggesting that microglia can become “primed” and display an activated phenotype chronically (Fenn et al., 2014; Witcher et al., 2015; Hoeijmakers et al., 2016; Ziebell et al., 2017). Microglia in the aged brain express “primed” morphology, even under healthy conditions, a facet not observed in the younger adult brain (Norden and Godbout, 2013; Loane et al., 2014). Hypotheses suggest priming allows microglia to respond more rapidly to injury and infection, however, the link between chronic microglial priming and neurodegeneration is under-explored (Norden and Godbout, 2013). Furthermore, other cells of the glial network also show a more reactive phenotype in the aging brain. Activation of astrocytes, with their physical role in maintaining the integrity of the BBB, may allow greater influx of soluble β-amyloid proteins into the brain (see section “Amyloid-β and Tau as Mediators of Inflammation and Sleep Disturbance”), thus advancing the pathologies of the disease.

Much like in AD, a primed microglial phenotype has been observed after TBI. One study in rodents showed that after TBI, microglia remained sensitized and quickly became activated after a peripheral immune challenge provided by lipopolysaccharide (LPS). Mice that had received a TBI and LPS appeared to have a poorer cognitive outcome, suggesting that microglial priming worsens cognition (Muccigrosso et al., 2016). Another study found that microglia displayed a primed morphology up to a year after controlled cortical impact which appeared to augment pathologies such as lesion volume, degeneration of hippocampal neurons and myelin loss, which suggests microglial priming may be, at least in part, a contributor to neurodegeneration post-TBI (Loane et al., 2014). However, this relationship is hard to pinpoint as not only do microglia express a primed morphology with age and TBI, but also with other inflammatory-associated conditions such as HIV (Hains et al., 2010), stroke (Espinosa-Garcia et al., 2017), and even exposure to air pollution (Mumaw et al., 2016).

After TBI, activated microglia have an increase in the number of lysosomes (Tanaka et al., 2013). This has been suggested to provide a link to the development of amyloid-β plaques, because of the acidic lysosomal pH in comparison with the extracellular microenvironment (Spangenberg et al., 2019). This work was continued using histological staining in human tissue and found an association between microglia containing amyloid-β-aggregates which are hypothesized to lead to progressive amyloid-β pathology (Spangenberg et al., 2019). To further demonstrate the role of microglia in plaque formation, elimination of microglia in a transgenic mouse model of AD eliminated plaque formation, but plaques returned with the recovery of microglia, which supports them having a role in plaque formation.

Eliminating and repopulating microglia have shown therapeutic efficacy in both AD and TBI. Eliminating microglia using a bioavailable colony stimulating factor 1 receptor (CSF1R) inhibitor, a receptor necessary for microglia survival, in a 5xFAD mouse model of AD showed amyloid-β plaques failed to form in the absence of microglia but when the CSF1R inhibitor was withdrawn, microglia repopulated and amyloid-β plaques developed similar to those in controls (Spangenberg et al., 2019). Similarly, the elimination of microglia with a low dose of a bioavailable CSF1R inhibitor in 3xTg-AD mice prevented microglia association with plaques and improved cognitive function (Dagher et al., 2015). Another study showed chronic microglia elimination did not alter amyloid-β loads but prevented neuronal loss, rescued dendritic spine loss, and improved cognitive behavior (Spangenberg et al., 2016). Microglia elimination has been used in experimental TBI studies where microglia elimination with a CSF1R inhibitor prevented acute and chronic inflammatory responses in the mouse and attenuated astrogliosis (Witcher et al., 2018). As a therapeutic intervention, chronically activated microglia were eliminated using a CSF1R inhibitor 1 month following a focal TBI, and the inhibitor was withdrawn 1 week later to allow microglia to repopulate. These studies indicated that removal of microglia after TBI reduced chronic neuroinflammation and associated neurodegeneration and mice had improved functional outcome (Henry et al., 2020). Taken together, these studies support that microglia play a role the chronic inflammatory response following TBI and contribute to AD pathology. If eliminating microglia changes sleep profiles in the context of TBI or AD has yet to be explored, however, these experiments may offer further therapeutic approaches to mitigate inflammation-mediated sleep disturbances.

## Conclusion

This review documents the complexity of the multidirectional relationship between sleep, microglia, and inflammation in the clinical and pre-clinical contexts of TBI and AD. It is posited that the inflammatory cascades triggered by the disturbed sleep observed in TBI and AD creates the perfect storm, resulting in a highly dynamic inter-disease trajectory. To this end, the proposed sleep and inflammation-mediated link between TBI and AD presents an opportunity for a multifaceted approach to clinical intervention.

## Author Contributions

TG and RR are responsible for the conceptualization and organization of this review. TG wrote the first draft of the manuscript. JO, SW, RW, and RR contributed to content and edited the manuscript. All authors contributed to the article and approved the submitted version.

## Conflict of Interest

The authors declare that the research was conducted in the absence of any commercial or financial relationships that could be construed as a potential conflict of interest.
